# Somatic manifestation of distress: clinical medicine, psychological, and public health perspectives

**DOI:** 10.1186/s13030-017-0119-3

**Published:** 2017-12-18

**Authors:** Mutsuhiro Nakao

**Affiliations:** 1 Teikyo University Graduate School of Medicine, Tokyo, Japan; 2 Division of Psychosomatic Medicine, Teikyo University Hospital, Tokyo, Japan

Somatic complaints are often related to chronic stress-related illnesses, but the diagnoses are not obvious in all cases. In some cases, patients could be classified with functional somatic syndromes. These syndromes are defined as several related disease-conditions that are characterized more by symptoms, suffering, and disability than by structural or functional abnormalities [1]. Functional somatic syndromes typically include irritable bowel syndrome, tension type headache, chronic fatigue syndrome, fibromyalgia, and some types of menstruation-related problems, all of which are frequently observed in psychosomatic medicine clinics. In addition, somatic complaints that are not fully explained by a medical condition could be diagnosed as somatic symptom disorders from a psychiatric perspective. According to the Diagnostic and Statistical Manual of Mental Disorders, Fifth Edition, the symptoms must cause clinically significant distress or impairment in social, occupational, or other areas of functioning [2].

In this thematic series of “somatic manifestation of distress: functional somatic syndromes and somatic symptom disorders”, a total of five articles [3–7] have been published to show somatic manifestations of distress from clinical medicine, psychological, and public health perspectives (Table 1). First, concepts such as somatization, somatosensory amplification, and somatosensory catastrophizing are introduced to explain somatic manifestations of distress [3]. Somatosensory amplification occurs when these bodily sensations become stronger and more painful. Somatosensory catastrophizing is the tendency to attribute these bodily sensations to unbearable functional modulation or as signs of serious illness. In this study, the close relationship between somatosensory amplification, somatosensory catastrophizing, and physical symptoms was clarified, and it was shown that somatizing patients exhibited significantly more somatosensory amplification and somatosensory catastrophizing, compared with those in the non-patient group.

**Table 1:** Outline of thematic series of "somatic manifestation of distress: functional somatic syndromes and somatic symptom disorders"

Clinical assessment:
Theoretical model of somatosensory amplification and somatosensory catastrophizing thinking [3]
Clinical epidemiology of functional somatic syndromes in psychosomatic medicine clinic [4]
Psychological intervention:
Autogenic training in functional somatic syndromes [5]
Brief internet-based cognitive behavior therapy for self-care [6]
Public health issue:
Physical change related to menstrual cycle in working women [7]

The clinical characteristics of somatizing patients have been summarized in the setting of a psychosomatic clinic at a Japanese university hospital [4]. Among approximately 600 new patients who had somatic symptoms at the psychosomatic clinic, 11% met the definition of functional somatic symptoms, and 74% had somatization associated with a mood or anxiety disorder. In that study, it was important to find statistically significant and positive tendencies between somatization and referral by a primary care physician because it suggests that consultation with a primary care physician may facilitate appropriate care at a psychosomatic clinic [8, 9].

Autogenic training, a treatment technique used to elicit a relaxation response, was performed in 24 patients with functional somatic syndromes who visited the outpatient clinic or were hospitalized at the department of psychosomatic medicine at a Japanese university hospital [5]. The autogenic training program consisted of three teaching sessions at intervals of 2 months and self-exercises for 3 min, twice a day. The program eased dysregulation of the autonomic nervous system, which was measured by the salivary amylase stress marker. In addition, the program contributed to decreasing tension-anxiety and somatic symptoms in patients with functional somatic syndromes. According to previous results of a meta-analysis on autogenic training [10], positive treatment effects have been identified for a variety of disease conditions, such as tension headache/migraine, mild-to-moderate essential hypertension, coronary heart disease, bronchial asthma, somatoform pain disorder (unspecified type), Raynaud’s disease, and functional sleep problems. Thus, the relaxation response techniques, including autogenic training, are expected to be effective in treating functional somatic syndromes.

As a promising psychological intervention of somatization, internet-based cognitive behavioral therapy has been reported to have a treatment effect on somatic symptoms in association with improvements in anxiety and other psychological conditions [6]. Internet-based cognitive behavior therapy is a technology-based technique delivered with or without the support of a clinician, and a previous meta-analysis indicated that computer-based psychological treatments are effective for depression and anxiety disorders [11]. Internet-based cognitive behavioral therapy may contribute to increased savings in indirect healthcare costs, due to the greater number of self-supporting functional patients with somatic syndrome [12].

Physical changes related to the menstrual cycle have been associated with excessive working hours in approximately 1600 female alumnae who graduated from a famous women’s university [7]. That study showed that a short time interval increases both anxiety and dissatisfaction with health in working women. According to a previous study that predicted functional somatic syndromes [13], female sex and a high level of intelligence as well as high numbers of functional somatic symptoms are significantly associated with the diagnosis of functional somatic syndrome. An unhealthy lifestyle is closely related to functional somatic symptoms or suboptimal health status [14], and increased efforts to modify unhealthy lifestyles, which include longer work times with shorter interval times, are necessary to prevent such disease conditions.

These five articles [3–7] will facilitate researchers’ study of biopsychosocial aspects of psychosomatic illnesses, functional somatic syndromes, and any stress-related health condition.
